# Assessment of Risk of Harm Associated With Intensive Blood Pressure Management Among Patients With Hypertension Who Smoke: A Secondary Analysis of the Systolic Blood Pressure Intervention Trial

**DOI:** 10.1001/jamanetworkopen.2019.0005

**Published:** 2019-03-08

**Authors:** Joseph Scarpa, Emilie Bruzelius, Patrick Doupe, Matthew Le, James Faghmous, Aaron Baum

**Affiliations:** 1 Icahn School of Medicine at Mount Sinai, New York, New York; 2 Arnhold Institute for Global Health, Icahn School of Medicine at Mount Sinai, New York, New York; 3 Department of Health System Design and Global Health, Arnhold Institute for Global Health, Icahn School of Medicine at Mount Sinai, New York, New York

## Abstract

**Importance:**

The randomized Systolic Blood Pressure Intervention Trial (SPRINT) showed that lowering systolic blood pressure targets for adults with hypertension reduces cardiovascular morbidity and mortality in general. However, whether the overall benefit from intensive blood pressure control masks important heterogeneity in risk is unknown.

**Objective:**

To test the hypothesis that the overall benefit observed in SPRINT masked important heterogeneity in risk from intensive blood pressure control.

**Design, Setting, and Participants:**

In this exploratory, hypothesis-generating, ad hoc, secondary analysis of data obtained from 9361 participants in SPRINT, a random forest–based analysis was used to identify potential heterogeneous treatment effects using half of the trial data. Cox proportional hazards regression models were applied to test potential heterogeneous treatment effects on the remaining data. The original trial was conducted at 102 sites in the United States between November 2010 and March 2013. This analysis was conducted between November 2016 and August 2017.

**Interventions:**

Participants were assigned a systolic blood pressure target of less than 120 mm Hg (intervention treatment) or of less than 140 mm Hg (standard treatment).

**Main Outcomes and Measures:**

The primary composite cardiovascular outcome was myocardial infarction, other acute coronary syndromes, stroke, heart failure, or death from cardiovascular causes.

**Results:**

Of 9361 participants in SPRINT, 466 participants (5.0%) were current smokers with systolic blood pressure greater than 144 mm Hg at baseline, with 230 participants (49.4%) randomized to the training data set and 236 participants (50.6%) randomized to the testing data set; 286 participants (61.4%) were male, and the mean (SD) age was 60.7 (7.2) years. Combinations of 2 covariates (ie, baseline smoking status and systolic blood pressure) distinguished participants who were differentially affected by the intervention. In the testing data, Cox proportional hazards models for the primary outcome revealed a number needed to harm of 43.7 to cause 1 event across 3.3 years among participants who, at baseline, were current smokers with systolic blood pressure greater than 144 mm Hg (10.9% [12 of 110] of primary outcome events for intervention treatment vs 4.8% [6 of 126] for standard treatment; hazard ratio, 10.6; 95% CI, 1.3-86.1; *P* = .03). This subgroup was also associated with a higher likelihood to experience acute kidney injury under intensive blood pressure control (with a frequency of 10.0% [11 of 110] of acute kidney injury events for intervention treatment vs 3.2% [4 of 126] for standard treatment; hazard ratio, 9.4; 95% CI, 1.2-77.3; *P* = .04).

**Conclusions and Relevance:**

In this secondary analysis of SPRINT data, current smokers with a baseline systolic blood pressure greater than 144 mm Hg had a higher rate of cardiovascular events in the intensive treatment group vs the standard treatment group. Further research is needed to evaluate the potential tradeoffs of intensive blood pressure control in hypertensive smokers.

## Key Points

**Question:**

Does clinically important heterogeneity exist in the risk of harm from intensive blood pressure control in adults with hypertension who smoke?

**Findings:**

In this ad hoc secondary analysis of the randomized Systolic Blood Pressure Intervention Trial (SPRINT), potential heterogeneous treatment effects were identified via a random forest–based analysis using half of the trial data and tested by applying Cox proportional hazards regression models to the remaining data. Smokers with systolic blood pressure greater than 144 mm Hg were associated with a significant increase in cardiovascular events from lower blood pressure targets despite overall positive trial findings.

**Meaning:**

Adults with hypertension who smoke may have a higher rate of cardiovascular events associated with intensive blood pressure control.

## Teaser

This secondary analysis of the Systolic Blood Pressure Intervention Trial uses a random forest–based analysis to assess whether clinically important heterogeneity exists in the risk of harm associated with intensive blood pressure treatment among adults with hypertension who smoke.

## Introduction

Hypertension is highly prevalent and a significant risk factor for death and disability-adjusted life years lost.^1^ The treatment of hypertension reduces the incidence of a number of cardiovascular-related events, including stroke, myocardial infarction, and heart failure.^2^ The Systolic Blood Pressure Intervention Trial (SPRINT) showed that treatment to a lower systolic blood pressure (SBP) target (<120 mm Hg) in adults without diabetes provides increased benefit over a more modest target (<140 mm Hg).^3^ These benefits were generally consistent with evidence from meta-analyses of randomized clinical trials for intensive blood pressure lowering,^2,4^ suggesting that intensive blood pressure management is broadly beneficial and may be an optimal treatment goal.

Many limitations of SPRINT have also been widely discussed, including the interpretability of its automated blood pressure measurements and the generalizability of its results to other hypertensive populations.^5^ Of note is that SPRINT, as well as the Action to Control Cardiovascular Risk in Diabetes Trial, also reported significant adverse events associated with intensive blood pressure control, including increased renal insufficiency and frequency of acute kidney injury episodes.^6,7^ These adverse events may limit the long-term adherence of intensive blood pressure control in practice, thereby reducing its practical efficacy.^8^

It is common that treatments have variable benefit and off-target effects in a population, but it is often difficult to identify subpopulations that are optimally targeted by an intervention. Traditional subgroup analyses typically fail to identify these heterogeneous treatment effects (HTEs) because the analyses are limited by multiple testing concerns, estimation bias, and prespecified univariate covariate testing,^9^ but recent statistical advances in machine learning permit detection of HTEs in large populations with many covariates.^10^ Identifying subgroups of patients who poorly respond to, or are at risk of harm from, treatment is essential for practicing clinicians to avoid the well-documented complications of intensive blood pressure interventions and thereby provide personalized, safe, and cost-effective treatment.^11^

In the present study, we use a machine learning method to investigate potential heterogeneity in the cardiovascular morbidity and mortality effects of intensive blood pressure control reported in SPRINT.^10^ The method adapts the machine learning technique random forest analysis to predict subgroup treatment associations over a rich set of variables and functional forms. Here, we construct a random forest in half of the trial data (training data) to identify patient subgroups that may have been harmed by intensive blood pressure control and then validate these hypotheses using Cox proportional hazards regression models in the remaining half of the data (testing data). We test the hypothesis that the overall benefit in the trial masked important heterogeneity in risk from intensive blood pressure control.

## Methods

This is an exploratory, hypothesis-generating, ad hoc, secondary analysis of data obtained from 9361 participants in SPRINT. Study design and reporting were based on the Transparent Reporting of a Multivariable Prediction Model for Individual Prognosis or Diagnosis (TRIPOD) reporting guideline, a standardized, evidence-based set of recommendations for reporting prediction modeling studies.^12^ The Icahn School of Medicine at Mount Sinai Institutional Review Board deemed this analysis exempt from review and waived the need for obtaining informed patient consent because the data were deidentified.

### Study Sample

The study sample consisted of all participants in SPRINT, a randomized, controlled, open-label trial assigning an SBP target of less than 120 mm Hg (intervention group) or assigning a target of less than 140 mm Hg (control group) to persons with an SBP of 130 mm Hg or higher and an increased cardiovascular risk. The study was conducted at 102 clinical sites in the United States between November 2010 and March 2013.^13^ Inclusion criteria included the following: age of at least 50 years, SBP between 130 and 180 mm Hg, and an increased risk of cardiovascular events (clinical or subclinical cardiovascular disease other than stroke; chronic kidney disease, excluding polycystic kidney disease, with an epidermal growth factor receptor of 20 to <60 mL per minute per 1.73 m^2^ of body surface area, calculated with the use of the 4-variable Modification of Diet in Renal Disease equation; a 10-year risk of cardiovascular disease of ≥15% on the basis of the Framingham risk score; or an age of ≥75 years). Patients with diabetes or prior stroke were excluded. After randomization, participant’s antihypertensive regimens were adjusted on the basis of the treatment assignment to achieve the appropriate systolic blood pressure (SBP) target. Demographic data were collected at baseline; clinical and laboratory data were obtained at baseline and every 3 months thereafter, with participants queried about medical events and hospitalizations every 6 months; and medical records and electrocardiograms were obtained for documentation of events, including serious adverse events. Full details of the original study methods and detailed inclusion and exclusion criteria are available elsewhere.^14^

### Outcomes and Predictors

In this post hoc secondary analysis, we used the SPRINT primary outcome as the trial’s primary composite cardiovascular outcome, which included myocardial infarction, other acute coronary syndromes, stroke, heart failure, or death from cardiovascular causes. Details on the 27 baseline factors we analyzed are provided in eTable 1 in the Supplement. Continuous variables were converted to discrete variables based on quartiles of their distribution.

### Statistical Analysis

In the present ad hoc analysis of the SPRINT cohort, the data of 9361 participants were randomly divided into 2 equal and independent subsets: a training set for machine learning–based hypothesis generation and a testing set for statistical inference–based hypothesis testing. We ensured that the distributions of covariates and outcomes in the training data were reflective of the whole data set using entropy weight minimization (eAppendix in the Supplement).^15^

Using the training data, we applied a random forest–based algorithm to identify subgroups with HTEs. The method identifies subgroups by building numerous nonparametric models from prespecified covariates to find the combinations of covariates that maximally explain the variation in outcomes across study groups. This strategy trains and validates models on different subsets of the data to calculate “honest” estimates and reduce overfitting. This approach therefore attempts to mitigate some of the concerns associated with multiple hypothesis testing and reduces the dangers of finding spurious correlations through a split-sample approach.^16^

We first constructed 1000 decision trees. In general, decision trees are designed to partition the sample into subgroups that share similar predictions or classifications. For each tree, 50% of the training data are randomly selected without replacement and are sequentially partitioned into covariate subgroups. The random forest–based algorithm we implemented was designed specifically for HTE prediction.^10^ For each level of a tree, the algorithm examines all possible split points for each covariate and selects covariate and split point pairs that minimize variation in the mean squared error of the predicted association between the treatment and outcome within each subgroup (leaf). The other 50% of the training data are used to estimate the association between the treatment and outcome in these subgroups, ensuring an “honest” estimate and reducing the possibility of overfitting (eAppendix in the Supplement). To generate testable hypotheses from the results of the forest, we identified all leaves (subgroups) with an estimated association between the treatment and outcome greater than zero, suggesting that the subgroup of patients in the leaf had higher rates of the primary outcome due to treatment. Six percent of all leaves met this criterion and were considered high-priority subgroup hypotheses to investigate in the test data.

In the testing data, these subgroup hypotheses were examined using Cox proportional hazards regressions to estimate hazard ratios (HRs) for the primary outcome, with stratification according to clinic site. Following standardized protocols for detection of HTEs, the Cox proportional hazards regression models contain terms for study group assignment, a subgroup dummy variable, and their interaction.^9^ To account for multiple hypothesis testing, we randomly permuted the subgroup assignment in the test data 1000 times. For each permutation, the Cox proportional hazards regression model was calculated with treatment, subgroup, and their interaction as independent covariates, stratified by clinic site, as used in the original test data. The false discovery rate (FDR) was estimated by calculating the proportion of the permuted interaction coefficients that were greater than the true interaction coefficient. A subgroup hypothesis was considered validated (eg, there was an adverse association between treatment and the primary outcome within that subgroup) only if (1) the HR for the interaction between the treatment and the subgroup was greater than 1 and statistically significant (2-tailed *P* < .05 and the FDR < 0.05) and (2) the HR for the subgroup was greater than 1 and statistically significant (2-tailed *P* < .05).

In addition to examining subgroup differences in the study’s primary composite cardiovascular outcome, we also tested whether the treatment was positively associated with adverse events that may have contributed to increased rates of the primary outcome within a subgroup, again using Cox proportional hazards regression models. All adverse outcomes available in the SPRINT data that were categorized as serious adverse events or resulted in emergency department visits were included in the analysis because these outcomes are interpretable and well documented.

To explore mechanisms through which the intervention may have contributed adversely to the study’s primary composite cardiovascular outcome, we analyzed blood pressure changes in the standard and intensive intervention groups during the course of the study. Differences between treatment groups were calculated for the means of the mean arterial pressure (calculated as ⅓ × [systolic blood pressure + (2 × diastolic blood pressure)]), diastolic pressure, and systolic pressure within the identified subgroup and among the rest of the cohort.

Data from SPRINT were obtained from the National Heart, Lung, and Blood Institute’s Biologic Specimen and Data Repository Information Coordinating Center.^17^ All analyses were performed between November 2016 and August 2017 using R, version 3.2.2 (R Foundation for Statistical Computing) and Stata, version 14 (StataCorp). The statistical code for the random forest analysis is available online.^18^

## Results

### Participants

Of 9361 participants in SPRINT, 466 participants (5.0%) were current smokers with SBP greater than 144 mm Hg at baseline, with 230 participants (49.4%) randomized to the training data set and 236 participants (50.6%) randomized to the testing data set; 286 participants (61.4%) were male, and the mean (SD) age was 60.7 (7.2) years. In the present ad hoc analysis, the SPRINT data set was randomly divided into a training set (n = 4681) and testing set (n = 4680). This training set was used for model development, while the testing data set was preserved for internal validation of hypotheses (Figure). The training sample included 2324 of 4678 participants randomized to the intervention group and 2357 of 4683 participants randomized to the control group. Demographic and relevant clinical characteristics of the included study sample are provided in Table 1. The participants included in the sample for model development had a mean (SD) age of 68 (9.4) years, a mean (SD) body mass index (calculated as weight in kilograms divided by height in meters squared) of 30.0 (5.8), and 1663 (35.5%) were female. Participants were followed up for a median (interquartile range) of 3.3 (2.7-3.8) years. A total of 122 of 2324 (5.2%) participants in the intervention group and 159 of 2357 (6.7%) participants in the control group experienced a primary outcome event.

**Figure.**
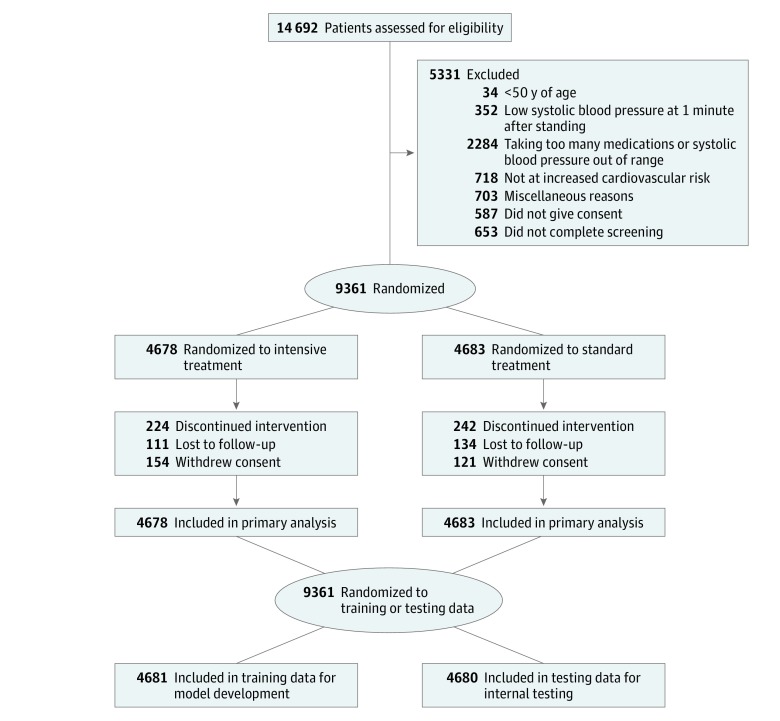
Flow of Patients in the Systolic Blood Pressure Intervention Trial and Through Randomized Partitioning Into Training and Testing Data Groups

**Table 1.** Baseline Clinical Characteristics of Patients Across Treated and Control Groups in the Overall Data Set, the Training Subset, and the Testing Subset

Baseline Covariate	Overall			Training Data			Testing Data		
	Mean (SD)		*P* Value	Mean (SD)		*P* Value	Mean (SD)		*P* Value
	Treated	Control		Treated	Control		Treated	Control	
Framingham estimation of 10-y CVD risk, points	20.1 (10.9)	20.2 (10.8)	.83	20.2 (10.5)	20.1 (10.9)	.22	20.1 (11.3)	20.2 (10.6)	.36
Current smoker, No. (%)	615 (13.3)	651 (13.9)	.27	296 (12.6)	323 (13.9)	.18	319 (13.7)	328 (13.9)	.83
Blood pressure, mm Hg									
Systolic	139.7 (15.7)	139.7 (15.5)	.96	140.0 (16.0)	139.5 (15.4)	.88	139.4 (15.5)	140.0 (15.7)	.94
Diastolic	78.2 (11.9)	78.1 (12.1)	.47	78.3 (12.2)	77.9 (11.9)	.77	78.2 (11.6)	78.3 (12.3)	.48
Daily aspirin use, No. (%)	2406 (50.8)	2350 (51.4)	.23	1208 (51.1)	1185 (52.0)	.24	1198 (50.5)	1165 (50.9)	.59
eGFR MDRD, mL/min/1.73m^2^	71.7 (20.7)	71.7 (20.7)	.92	71.5 (20.5)	71.1 (20.6)	.47	71.8 (20.9)	72.4 (20.8)	.56
Serum creatinine, mg/dL	1.1 (0.35)	1.1 (0.34)	.73	1.1 (0.34)	1.1 (0.34)	.27	1.1 (0.35)	1.1 (0.34)	.56
Black or African American, No. (%)	1493 (31.9)	1454 (31.1)	.40	754 (32.0)	744 (32.0)	.99	739 (31.8)	710 (30.2)	.23
Age at randomization top-coded at 90 y, y	67.9 (9.4)	67.9 (9.5)	.94	68.1 (9.4)	67.8 (9.4)	.50	67.7 (9.4)	68.0 (9.6)	.43
Female, No. (%)	1648 (35.2)	1684 (36.0)	.41	804 (34.1)	859 (37.0)	.04	844 (36.3)	825 (35.1)	.38
Cholesterol, mg/dL	190.4 (40.7)	189.6 (40.6)	.85	190.0 (41.9)	190.3 (41.4)	.66	190.8 (41.5)	188.8 (39.7)	.87
Glucose, mg/dL	98.9 (13.8)	98.9 (13.4)	.86	98.9 (13.3)	98.8 (13.7)	.68	98.9 (14.3)	99.0 (13.1)	.51
HDL-C direct, mg/dL	52.9 (14.4)	52.7 (14.6)	.66	52.6 (14.2)	52.7 (15.0)	.54	53.1 (14.3)	52.8 (13.1)	.20
Triglycerides, mg/dL	125.6 (87.1)	126.6 (80.9)	.22	125.3 (92.2)	127.1 (83.3)	.54	125.8 (81.6)	126.1 (78.4)	.24
Urine albumin in mg/(creatinine in g × 0.01)	43.7 (178.4)	41.3 (154.2)	.39	43.0 (150.8)	41.8 (135.3)	.57	44.3 (202.2)	40.9 (171.1)	.52
BMI	29.9 (5.8)	29.8 (5.74)	.39	30.0 (5.9)	29.9 (5.6)	.14	29.9 (5.8)	29.7 (5.9)	.77
Receiving any statin, No. (%)	2076 (44.3)	1978 (42.3)	.04	1065 (45.2)	986 (42.4)	.05	1011 (43.5)	992 (42.1)	.32

Abbreviations: BMI body mass index (calculated as weight in kilograms divided by height in meters squared); CVD, cardiovascular disease; eGFR, epidermal growth factor receptor; HDL-C, high-density lipoprotein cholesterol; MDRD, Modification of Diet in Renal Disease.

SI conversion factors: To convert serum creatinine to micromoles per liter, multiply by 88.4; glucose to millimoles per liter, multiply by 0.0555; triglycerides to millimoles per liter, multiply by 0.113; and total cholesterol and HDL-C to millimoles per liter, multiply by 0.0259.

### Model Development

Using the training data, the random forest algorithm identified subgroups with high likelihood of differential benefit or harm from the intensive blood pressure intervention. There were a total of 5250 leaves in the 1000 trees tested in the random forest. In the majority of leaves (4911 [94%]), the estimated association between treatment and the primary outcome indicated variable benefit from the intervention, but the remaining 339 leaves (6%) indicated potential harm. Relative to all other covariates, those associated with kidney function (serum creatinine and albumin to creatinine ratio) and cardiovascular disease risk factors (10-year Framingham risk score, total cholesterol, and smoking status) most frequently defined membership in the 6% of subgroups with a higher likelihood of being adversely affected by treatment (eTable 2 in the Supplement).

### Internal Testing

Using the testing data, we investigated the subset of HTE hypotheses identified by the random forest analysis as indicating potential harm. The testing data set included 2354 of 4678 participants randomized to the intervention group and 2326 of 4683 participants randomized to the control group. Analyses of the other participant characteristics (Table 1) validated that the covariates were balanced across training and testing data in general, as well as between control and intervention groups within each data subset (see eAppendix in the Supplement).

Although the testing cohort as a whole was associated with benefit from treatment (HR, 0.7; 95% CI, 0.5-0.9; *P* < .05), our analysis validated that 1 subgroup (ie, current smokers with a baseline SBP >144 mm Hg) had a significantly greater frequency of primary events in the treatment group (10.9% [12 of 110]) than in the control group (4.8% [6 of 126]), with an HR of 10.6 (95% CI, 1.3-86.1; *P* = .03) (Table 2). The number needed to harm for this subgroup was 43.7 to cause 1 event. The HR for the interaction between treatment and subgroup membership was 3.5 (95% CI, 1.2-9.6; *P* = .02) and remained significant when accounting for multiple comparisons (FDR < 0.05) (eFigure 2 in the Supplement). Further analyses of the separate effects of baseline current smoking and SBP greater than 144 mm Hg revealed HRs that were not significant either among current smokers overall or among all participants with an SBP greater than 144 mm Hg at baseline (eTable 3 in the Supplement). Results of the full 3-way interaction model suggested that the interaction of smoking status and SBP was key to the observed heterogeneity, with only the HR on the triple interaction term (treated × baseline current smoker × baseline SBP >144 mm Hg) being significant (HR, 2.0; 95% CI, 1.1-3.7; *P* < .05) (eTable 3 in the Supplement). Besides the subgroup of participants who were hypertensive and smoked, no other subgroup analyzed had an HR greater than 1 with *P* < .05.

**Table 2.** Observed Outcomes by Treatment Group Using Testing Data, Stratified by Subgroup Identified Using Training Data

Primary Composite Outcome^a^	No. of Patients (No. of Events)		HR (95% CI)	*P* Value
	Treated	Control		
Overall	2354 (121)	2326 (160)	0.7 (0.6-0.9)	.02
Subgroups				.02^b^
Current smokers with baseline systolic blood pressure >144 mm Hg	110 (12)	126 (6)	10.6 (1.3-86.1)	.03
Remainder of trial population	2244 (109)	2200 (154)	0.7 (0.5-0.9)	.003

Abbreviation: HR, hazard ratio.

^a^ Includes myocardial infarction, other acute coronary syndromes, stroke, heart failure, or death due to cardiovascular causes.

^b^ *P* value for the interaction term; false discovery rate for the interaction term is 0.007.

### Exploratory Analysis of Adverse Events and Mechanisms

Further analyses indicated that acute kidney injury, a treatment-associated serious adverse event, was significantly more prevalent in current smokers with a baseline SBP greater than 144 mm Hg. This subgroup of participants was more likely to experience acute kidney injury under intensive blood pressure control (10.0% [11 of 110]) compared with usual care (3.2% [4 of 126]) (for current smokers with high blood pressure: HR, 9.4; 95% CI, 1.2-77.3; *P* = .04; for all remaining participants: HR, 1.6; 95% CI, 1.2-2.2; *P* = .005) (eTable 4 in the Supplement). Participants in this subgroup who were randomized to treatment were also more likely to experience acute kidney injury than treated participants who were not in this subgroup (5.4%, *P* = .007).

To investigate potential mechanisms through which the subgroup experienced both a greater number of primary events and serious adverse acute kidney injury events in response to treatment, longitudinal changes in SBP and diastolic blood pressure were studied. As expected, both standard and intensive treatment reduced mean arterial blood pressure during the course of the trial among the subgroup and all remaining participants. However, the reduction in mean arterial pressure was greater in the hypertensive smoker subgroup than in the rest of the cohort (−8.7; 95% CI, −15.0 to −2.3; *P* = .01), driven by a far more sustained and pronounced reduction in diastolic blood pressure (approximately 2-fold) than SBP (eAppendix and eFigure 1 in the Supplement).

## Discussion

This exploratory, hypothesis-generating secondary analysis of SPRINT data revealed clinically meaningful heterogeneity associated with intensive blood pressure control in SPRINT and identified a subgroup of individuals who may have been harmed by an intervention that had an overall beneficial effect. Specifically, current smokers with a baseline systolic blood pressure greater than 144 mm Hg had a significantly higher rate of the primary composite outcome in the intensive treatment group compared with the standard treatment group. The patients in the intensive treatment group appeared to have experienced significant increases in the incidence of acute kidney injury and a significant and sustained reduction in diastolic blood pressure.

These findings are consistent with descriptive evidence from the Hypertension Optimal Treatment study, which also suggested that aggressive diastolic blood pressure lowering in smokers may increase risk for cardiovascular events.^19,20^ Because both smoking and hypertension have harmful effects on high-pressure vasculature, it has been suggested that patients with both risk factors may be more sensitive to a significant reduction in perfusion pressure.^21^ Lowering diastolic blood pressure in other cohorts with reduced arterial compliance and elasticity, such as elderly hypertensive populations, has also been noted to induce harm.^22,23^ Further research is needed to directly evaluate intensive blood pressure control in hypertensive smokers to better characterize the potential tradeoffs in this population. If the treatment effect heterogeneity identified in the present study holds within the general US population, it would forecast an additional 88 700 cases per year of acute kidney injury and 56 100 cases per year of hypotension should the SPRINT intensive blood pressure regimen be adopted.^24^

In addition, these findings suggest a potential mechanism for treatment harm in intensive blood pressure control that may provide insight for reducing risks associated with the intervention in practice. Previous studies have indicated that maintaining diastolic perfusion pressure is important to preventing acute kidney injury in the acute setting.^25^ Further evidence suggests that diastolic blood pressure affects mortality in patients with acute kidney injury.^26^ Our findings provide additional, suggestive evidence that diastolic blood pressure regulation may play an important role in reducing the risk of serious adverse events.

### Limitations

Despite these potentially relevant findings, the present study also had several important limitations that require careful consideration, and its findings must be interpreted as hypothesis generating. First, the random forest–based analysis we used splits the data into training and testing sets, using the training data to generate high-confidence hypotheses through machine learning techniques and then confirming or refuting these hypotheses in the testing set with traditional Cox proportional hazards regression modeling. While this strategy is conservative when compared with entirely exploratory data-driven approaches, it remains possible that the results we observed were spurious, that is, due to chance alone as a result of the idiosyncratic split. In an attempt to mitigate this effect, we calculated entropy weights for training and testing sets after 1000 different random splits to select a final training and testing data set with comparable covariate variance. Further testing and replication in other samples are required for validation. In addition, our cutoff of an SBP greater than 144 mm Hg for subgroup membership was not based on a clinically meaningful construct or ex ante hypothesis. Rather, it derived from discretization of the continuous SBP variable, reflecting the top tercile of participant SBP. We attempted to enhance our confidence in the observed results by further examining potential mechanisms, confirming that participants in this subgroup experienced increased rates of acute kidney injury and decreased diastolic blood pressure. Coupled with similar findings from the Hypertension Optimal Treatment study and other observational work, the findings suggest that further research is needed to better characterize the risks and benefits of intensive blood pressure control in hypertensive smokers.

A second related limitation is that, by splitting the data, we significantly reduced our power to detect meaningful differences in the hazards of the primary outcome in the testing data. The modest statistical power may have partly contributed to the wide CIs we observed. It is also likely that bona fide subgroups could be missed by this method. Besides the subgroup of hypertensive smokers, no other subgroup analyzed had an HR greater than 1 with *P* < .05, and only 1 subgroup (participants with baseline glucose levels in the bottom quartile and with a urine albumin to creatinine ratio in the bottom half of the distribution) had an HR greater than 1 with *P* < .10 (eTable 5 in the Supplement). However, by randomly splitting the data into independent halves, we were able to use the training data to select considerably fewer hypotheses to test in the testing data, thereby compensating for the loss of power from halving the sample size by reducing the size of the correction required to counteract multiple hypothesis testing.^16^ Similarly, estimating an FDR is a challenge given covariate dependencies in hypothesized subgroups. Nested FDR approaches may be one way to recover some power when testing multiple hypotheses, but more formal study is required to identify optimal ways to minimize multiple hypothesis testing error when searching a multicovariate space for heterogeneous treatment effects. Another potential concern is that censoring was slightly greater among the hypertensive smoker subgroup members than among the remaining participants. It is important to note that the random forest–based algorithm we used does not account for censoring when identifying subgroup hypotheses in the training data. This limitation was addressed by using Cox proportional hazards regression models in the testing data, but the covariate partitioning algorithm can be improved by including censoring explicitly in the construction of each tree.

## Conclusions

This post hoc secondary analysis of SPRINT data demonstrates that a machine learning method for HTE prediction can reveal unexpected and previously unappreciated HTEs within an existing randomized clinical trial to identify potential hypotheses to test in future research. Understanding heterogeneity in treatment effects is critically important so that clinicians can target an intervention to patients most likely to benefit and can avoid unnecessary harm to hidden high-risk groups. Further prospective study is required to determine how different blood pressure targets may harm high-risk subgroups, such as hypertensive smokers. Greater mechanistic evidence for the role of current smoking status on aggressive SBP lowering and careful investigation of reliable indicators of risk, including diastolic blood pressure, are also warranted. Our findings suggest that randomized clinical trials with beneficial effects can still have unwanted effects in population subgroups. Estimating HTEs with data-driven methods can be an important step in safely and rationally applying scientific insight to clinical practice.

## Notes

Supplement. **eAppendix.** Details on Underlying Functioning of Causal Forest Method **eTable 1.** Twenty-Seven Baseline Predictors **eTable 2.** Covariates That Most Frequently Defined the 6% of Subgroups (Leaves) Identified in the Training Data as Having a Higher Likelihood of Being Adversely Affected by Treatment **eTable 3.** Observed Outcomes by Treatment Group Using Testing Data for Current Smokers, Participants With Baseline Systolic Blood Pressure (SBP) > 144 mmHg, and their Interaction **eTable 4.** Observed Serious Adverse Event Outcomes by Treatment Group Using Testing Data, for the Subgroup Identified Using Training Data **eTable 5.** Observed Outcomes by Treatment Group Using Testing Data for Subgroups Defined by a Baseline Glucose in the Bottom Quartile (<91 mg/dl), Urine Albumin/Creatinine Ratio in the Bottom Half (<9.5 mg/g Cr) of the Distribution, and Their Interaction **eFigure 1.** Blood Pressure Changes Across Treatment Group for Hypertensive Smokers vs. Remaining Participations **eFigure 2.** Distributions of P-values and False Discovery Rates Across Subgroups Identified by the Causal Forest **eReferences.**
