# Incorrect Affiliation

**DOI:** 10.1001/jamanetworkopen.2019.3146

**Published:** 2019-04-05

In the Original Investigation titled “Assessment of Risk of Harm Associated With Intensive Blood Pressure Management Among Patients With Hypertension Who Smoke: A Secondary Analysis of the Systolic Blood Pressure Intervention Trial,”^1^ published March 8, 2019, the affiliation listed for Dr Faghmous was incorrect. The correct affiliation is Department of Medicine, Stanford University, Palo Alto, California. This article has been corrected.^1^
