# Supplementary material for: Assessment of Risk of Harm Associated With Intensive Blood Pressure Management Among Patients With Hypertension Who Smoke: A Secondary Analysis of the Systolic Blood Pressure Intervention Trial
Source: JAMA Netw Open. 2019 Mar 8;2(3):e190005. doi: 10.1001/jamanetworkopen.2019.0005 (PMC6484649; doi:10.1001/jamanetworkopen.2019.0005)
Supplement: Supplement. — eAppendix. Details on Underlying Functioning of Causal Forest Method eTable 1. Twenty-Seven Baseline Predictors eTable 2. Covariates That Most Frequently Defined the 6% of Subgroups (Leaves) Identified in the Training Data as Having a Higher Likelihood of Being Adversely Affected by Treatment eTable 3. Observed Outcomes by Treatment Group Using Testing Data for Current Smokers, Participants With Baseline Systolic Blood Pressure (SBP) > 144 mmHg, and their Interaction eTable 4. Observed Serious Adverse Event Outcomes by Treatment Group Using Testing Data, for the Subgroup Identified Using Training Data eTable 5. Observed Outcomes by Treatment Group Using Testing Data for Subgroups Defined by a Baseline Glucose in the Bottom Quartile (<91 mg/dl), Urine Albumin/Creatinine Ratio in the Bottom Half (<9.5 mg/g Cr) of the Distribution, and Their Interaction eFigure 1. Blood Pressure Changes Across Treatment Group for Hypertensive Smokers vs. Remaining Participations eFigure 2. Distributions of P-values and False Discovery Rates Across Subgroups Identified by the Causal Forest eReferences. [file jamanetwopen-2-e190005-s001.pdf]

Supplementary Online Content

Scarpa J, Bruzelius E, Doupe P, Le M, Faghmous J, Baum, A. Assessment of risk of harm associated with intensive blood pressure management among patients with hypertension who smoke: a secondary analysis of the Systolic Blood Pressure Intervention Trial. *JAMA Netw Open*. 2019;2(2):e190005. doi:10.1001/jamanetworkopen.2019.0005

eAppendix. Details on Underlying Functioning of Causal Forest Method

eTable 1. Twenty-Seven Baseline Predictors

eTable 2. Covariates That Most Frequently Defined the 6% of Subgroups (Leaves) Identified in the Training Data as Having a Higher Likelihood of Being Adversely Affected by Treatment

eTable 3. Observed Outcomes by Treatment Group Using Testing Data for Current Smokers, Participants With Baseline Systolic Blood Pressure (SBP) > 144 mmHg, and their Interaction

eTable 4. Observed Serious Adverse Event Outcomes by Treatment Group Using Testing Data, for the Subgroup Identified Using Training Data

eTable 5. Observed Outcomes by Treatment Group Using Testing Data for Subgroups Defined by a Baseline Glucose in the Bottom Quartile (<91 mg/dl), Urine Albumin/Creatinine Ratio in the Bottom Half (<9.5 mg/g Cr) of the Distribution, and Their Interaction

eFigure 1. Blood Pressure Changes Across Treatment Group for Hypertensive Smokers vs. Remaining Participations

eFigure 2. Distributions of P-values and False Discovery Rates Across Subgroups Identified by the Causal Forest

eReferences.

This supplementary material has been provided by the authors to give readers additional information about their work.

© 2019 Scarpa J et al. *JAMA Network Open*.

eAppendix: Details on Underlying Functioning of Causal Forest Method

Overview

This paper applies recent advances in machine learning for causal inference to conduct a post-hoc analysis of a randomized controlled trial (RCT). The Systolic Blood Pressure Intervention Trial (SPRINT) clinical trial we focus on demonstrated that treatment to a lower systolic blood pressure target (<120 mmHg) in non-diabetic adults provides increased benefit over a more modest target (<140mmHg)(1). However, we hypothesized that the positive average treatment effect may mask clinically- and policy-relevant heterogeneity.

Causally interpreting post-hoc analyses of RCTs is challenging because investigators may test a large number hypotheses, but only report those with significant treatment effects. On the other hand, the small set of pre-specified hypotheses registered ex-ante by investigators may leave clinically useful relationships between interventions, outcomes, and subgroups undiscovered. Recognizing the limitations of conventional approaches to subgroup analyses, and the fact that many clinical trials will be underpowered to detect meaningful treatment variation, a number of newer approaches to identifying heterogeneous treatment effect (HTEs) have been proposed.(2) These include a class of more data-driven predictive risk modeling tools such as Classification and Regression Trees which are typically most appropriate for early exploratory analyses.

The post-hoc analysis method we employ, called causal forest, extends classical recursive partitioning methods (e.g. random forest) to identify causally relevant subgroups defined by interactions of many variables, a combinatorial task for which human intuition and expertise is poorly suited. The initial, and conceptually important, step is to randomly split the data into two

© 2019 Scarpa J et al. *JAMA Network Open*.

independent halves, using the first partition for hypothesis generation/tree construction (training data) and preserving the remainder of the data for statistically valid inference (testing data). The method first identifies subgroups with similar treatment effects in the training data, then tests the most promising HTE hypotheses on the testing data to mitigate multiple testing concerns.

Partitioning the data

The SPRINT data (n=9,361) was randomly divided into two equal subsets: a training set for machine learning-based hypothesis generation and a testing set for statistical inference-based hypothesis testing. To ensure the training data was reflective of the whole data set, we constrained the split to guarantee the average treatment effect in the training data was within 1% of the originally reported overall hazard ratio for the primary outcome and covariate distributional balance, both across the training and testing data and between treated and control groups within each partition, using entropy weight minimization. Specifically, to select an optimal split, one thousand different random divisions of the full data were analyzed. To evaluate if the training data was reflective of the whole data set, the Cox proportional hazards regression was used to calculate the hazard ratio in the training data of each split. Splits with a training data hazard ratio +/- 1% of the originally reported hazard ratio (0.75) were further evaluated. For these splits, entropy weights were calculated to estimate the covariate balance between the training and testing data. The covariate distribution across treatment and control groups within the training data and the testing data, respectively, was also evaluated. For each split, the variance was calculated for these three weight vectors: the first compares the balance of covariates between the candidate training and testing data, the second compares the balance of covariates between the treatment groups of the candidate training and testing data, and the last

© 2019 Scarpa J et al. *JAMA Network Open*.

compares the covariate balance between the control groups. Each split was assigned three ranks, based on the variance of each weight vector. A composite rank was calculated for each data split by minimizing the variance of three weight vectors. and the optimal split was determined to be the one with the lowest composite rank. The final training data had 4,681 participants and the final testing data had 4,680 participants.

Identification of subgroups using the training data

To identify subgroups, we constructed an ensemble of causal trees(3), a type of decision tree. Decision trees are especially well-suited for identifying subgroups because they produce a partition of the sample in which subgroups share similar predictions or classifications that is not limited by model specification assumptions (as compared to several other approaches, e.g. (4) and (5)).In each causal tree, half the sample is randomly selected and its covariate space is sequentially partitioned into subspaces. Each split minimizes variation in the mean squared error of the estimated average treatment effect within each subspace. Because the structure of a single tree depends on the training data, different training data may yield vastly different trees. To account for the high variance in any given tree, an ensemble of trees (a “forest”) is often used. In this study, we constructed a forest of 1,000 trees.

Trees with an overall treatment effect within 1 median absolute deviation of the ensemble (“forest”) were prioritized for downstream analysis since they are likely the most robust and reproducible. To investigate subgroups with higher likelihood of being adversely affected by treatment, we identified all leaves with a positive average treatment effect, suggesting that the

© 2019 Scarpa J et al. *JAMA Network Open*.

subgroup[1] of patients in the leaf may have had higher mortality due to treatment. Six percent of all leaves met this criterion and were considered high-priority subgroup hypotheses to investigate in the testing data.

Estimating HTE using the testing data

For these subgroup hypotheses, a Cox proportional hazards regression was used to estimate the significance of the hazard ratio for the primary outcome, with stratification according to clinic site. Following standardized protocols for detection of HTEs, the Cox models contain terms for study-group assignment, a subgroup dummy variable, and their interaction. To account for multiple hypothesis testing, we randomly permuted the subgroup assignment in the test data 1,000 times. For each permutation, the cox model was calculated with treatment, subgroup, and their interaction as independent covariates, stratified by clinic site, as employed in the original test data. The false discovery rate (FDR) was estimated by calculating the proportion of the permuted interaction coefficients that were greater than true interaction coefficient. A subgroup was considered adversely affected only if (i) the hazard ratio for the interaction between the treatment and the subgroup was greater than 1 and significant ($p < 0.05$ and the false discovery rate (FDR) < 0.05) and (ii) the hazard ratio for the subgroup was greater than 1 and significant ($p < 0.05$)

[1] Subgroups (leaves) were defined by covariate values exclusive of the split points, e.g. ≤ 6 was tested as < 6.

© 2019 Scarpa J et al. *JAMA Network Open*.

eTable 1. Twenty-Seven Baseline Predictors

Variable	Label	Source	Value	Description
risk10yrs	Derived: Framingham estimation of 10-year CVD risk	Inclusion/Exclusion Summary; Central Laboratory (no form)	numeric value	Computed 10-year risk of CVD based on Framingham risk equation
InclusionFRS	Derived: Framingham 10-year CVD risk >15%	Inclusion/Exclusion Summary; Central Laboratory (no form)	0 - No; 1 - Yes	0/1 indicator whether 10-year Framingham risk score is >15%
sbp	Derived: Seated Systolic Blood Pressure (mm Hg)	BP Mangement Baseline; Inclusion/Exclusion	numeric value	Seated SBP from Baseline BP Management Form or Inclusion/Exclusion Summary if missing on BP Management
dbp	Derived: Seated Diastolic Blood Pressure (mm Hg)	BP Mangement Baseline; Inclusion/Exclusion	numeric value	Seated DBP from Baseline BP Management Form or Inclusion/Exclusion Summary if missing on BP Management
n_agents	Derived: Number of medications prescribed	Blood Pressure Medication Management Log	numeric value	Number of distinct anti-hypertensive agents prescribed at baseline visit (prior to randomization)
noagents	Derived: Participants on no anti-hypertensive agents	Blood Pressure Medication Management Log	0 - one or more agents; 1 - on no agents	0/1 indicator whether participant on NO anti-hypertensives at baseline visit (prior to randomization)
smoke_3cat	Derived: Baseline smoking status	Self-Administered Baseline History	1 - Never; 2 - Former; 3 - Current; 4 - Missing	categorization of smoking status from Tobacco Questionnaire (questions 51-53) on Self-administered Baseline History
aspirin	BSL Hist: Daily Aspirin Use	Self-Administered Baseline History	0 - No; 1 - Yes	question 56 on Self-administered Baseline History
egfr	Lab: eGFR MDRD (mL/min/1.73m^2)	Central Laboratory (no form)	numeric value	estimated glomerular filtration rate (eGFR) from baseline blood draw
screat	Lab: serum creatinine, mg/dL	Central Laboratory (no form)	numeric value	serum creatinine from baseline blood draw
sub_ckd	Derived: Subgroup with CKD (eGFR<60)	Central Laboratory (no form)	0 - No; 1 - Yes	participants with baseline eGFR <60 mL/min/1.73m^2 assigned value of "1"; all others (including those missing baseline eGFR assigned value of "0"
race_black	Incl/Excl: Black, African-American	Inclusion/Exclusion Summary	0 - No; 1 - Yes	0/1 indicator of African American race by self-report
age	Derived: Age at randomization top-coded at 90 years	Inclusion/Exclusion Summary	numeric value	calculated from date of birth on Inclusion/Exclusion summary and top-coded at 90 years

© 2019 Scarpa J et al. *JAMA Network Open*.

female	Derived: Age at randomization top-coded at 90 years	Inclusion/Exclusion Summary	0 - Male; 1- Female	0/1 indicator of female gender
sub_cvd	Derived: subgroup with history of clinical/subclinical CVD	Inclusion/Exclusion Summary	0 - No; 1 - Yes	0/1 indicator of history of one or more of MI, ACS, coronary revascularization, carotid revascularzation, PAD with revascularization, >50% stenosis of coronary/carotid/lower extremity artery; AAA ≥5 mm, coronary arter calcium score ≥400, ABI ≤0.90, or LVH
sub_clinicalcvd	Derived: subgroup with history of clinical CVD	Inclusion/Exclusion Summary	0 - No; 1 - Yes	0/1 indicator of history of one or more of MI, ACS, coronary revascularization, carotid revascularzation, PAD with revascularization, >50% stenosis of coronary/carotid/lower extremity artery; or AAA ≥5 mm,
sub_subclinicalcvd	Derived: subgroup with history of subclinical CVD	Inclusion/Exclusion Summary	0 - No; 1 - Yes	0/1 indicator of history of one or more of coronary arter calcium score ≥400, ABI ≤0.90, or LVH
sub_senior	Derived: subgroup ≥75 years old at randomization	Inclusion/Exclusion Summary	0 - No; 1 - Yes	0/1 indicator of age ≥75 at randomization
race4	Derived: Four-level race variable (character)	Inclusion/Exclusion Summary	HISPANIC, BLACK, WHITE, OTHER	self-reported race/ethnicity, if Hispanic ethnicity then value is "HISPANIC" all other values are non-Hispanic
CHR	Lab: Cholesterol, mg/dL	Central Laboratory (no form)	numeric value	total cholesterol from baseline blood draw
GLUR	Lab: Glucose, mg/dL	Central Laboratory (no form)	numeric value	serum glucose from baseline blood draw
HDL	Lab: HDL-cholesterol direct, mg/dL	Central Laboratory (no form)	numeric value	HDL-cholesterol from baseline blood draw
TRR	Lab: Triglycerides, mg/dL	Central Laboratory (no form)	numeric value	Triglycerides from baseline blood draw
UMALCR	Lab: mg Urine Alb / (g Creat * 0.01), mg/g Cr	Central Laboratory (no form)	numeric value	urine albumin/creatinine ratio from baseline sample, measured values <2 mg/g coded as missing
BMI	Derived: body mass index (kg/m^2)	Baseline Medications and Physical Exam	numeric value	body mass index (kg/m^2) calculated from recorded weight and height
statin	Derived: on any statin	Baseline Medications and Physical Exam	0 - No; 1 - Yes	0/1 indicator based on concomitant medications reported at baseline
SBPTertile	Derived: Systolic BP tertile	BP Mangement Baseline; Inclusion/Exclusion	1: <=144; 2: >144-<145; 3: >=145	baseline SBP divided into three groups based on tertiles (equal thirds) of the empirical distribution

© 2019 Scarpa J et al. *JAMA Network Open*.

eTable 2. Covariates That Most Frequently Defined the 6% of Subgroups (Leaves) Identified in the Training Data as Having a Higher Likelihood of Being Adversely Affected by Treatment

**Covariate**	**Frequency**
Framingham estimation of 10-year CVD risk	67
Cholesterol (mg/dL)	66
mg Urine Alb / (g Creat * 0.01), mg/g Cr	63
Baseline smoking status	54
Serum creatinine (mg/dL)	40
Diastolic Blood Pressure (mm Hg)	34
Body mass index (kg/m^2)	33
eGFR MDRD (mL/min/1.73m^2)	33
HDL-cholesterol direct (mg/dL)	28
Systolic Blood Pressure (mm Hg)	27
Systolic Blood Pressure Percentile	27
Glucose(mg/dL)	26
Triglycerides (mg/dL)	25
Race (HISPANIC, BLACK, WHITE, OTHER)	21
Female	16
≥75 years old at randomization	13
Daily Aspirin Use	9
On any statin	9
History of clinical/subclinical CVD	3

© 2019 Scarpa J et al. *JAMA Network Open*.

eTable 3. Observed Outcomes by Treatment Group Using Testing Data for Current Smokers, Participants With Baseline Systolic Blood Pressure (SBP) > 144 mmHg, and their Interaction

Panel A. Results of Subgroup Models	**Hazard Ratio on Treatment**	
	**HR [95% CI]**	**P value**
**Subgroup**		
Current Smokers	1.65 [0.84-3.26]	0.148
SBP greater > 144 mmHg	0.75 [0.51-1.10]	0.144
Current smokers with SBP > 144 mmHg	10.56 [1.29-86.13]	0.028
Panel B. Results of Interaction Models	**Hazard Ratio on Interaction Term**	
	**HR [95% CI]**	**P value**
**2-Way Interaction Model: Current Smoker**		
Current Smoker × Treatment	2.18 [1.12-4.21]	0.021
**2-Way Interaction Model: SBP>144mmHg**		
SBP>144 mmHg × Treatment	0.91 [0.56-1.48]	0.702
**3-Way Interaction Model**		
Current Smoker × Treatment	1.14 [0.65-1.99]	0.654
SBP>144 mmHg × Treatment	0.83 [0.55-1.24]	0.353
Current Smoker × SBP>144 mmHg × Treatment	1.99 [1.07-3.71]	0.030

© 2019 Scarpa J et al. *JAMA Network Open*.

eTable 4. Observed Serious Adverse Event Outcomes by Treatment Group Using Testing Data, for the Subgroup Identified Using Training Data

	**No. patients in Subgroup (No. of events)**		**Hazard Ratio**	
**Serious Adverse Event**	**Treated**	**Control**	**HR [95% CI]**	**P value**
AKI or ARF ER Visit or SAE event	110 (11)	126 (4)	9.44 [1.15-77.29]	p=0.036
Hypotension ER Visit or SAE event	110 (7)	126 (3)	7.13 [0.75-67.8]	p=0.087
Syncope ER Visit or SAE event	110 (7)	126 (4)	4.06 [0.44-37.67]	p=0.218
Electrolyte abnormality ER Visit or SAE event	110 (8)	126 (4)	3.55 [0.69-18.22]	p=0.128
Injurious fall ER Visit or SAE event	110 (6)	126 (5)	1.59 [0.36-7.11]	p=0.540
Orthostatic Hypotension with dizziness event	110 (2)	126 (4)	0.39 [0.03-4.44]	p=0.447
Orthostatic Hypotension event without dizziness	110 (11)	126 (34)	0.22 [0.06-0.75]	p=0.022

© 2019 Scarpa J et al. *JAMA Network Open*.

eTable 5. Observed Outcomes by Treatment Group Using Testing Data for Subgroups Defined by a Baseline Glucose in the Bottom Quartile (<91 mg/dl), Urine Albumin/Creatinine Ratio in the Bottom Half (<9.5 mg/g Cr) of the Distribution, and Their Interaction

Panel A. Results of Subgroup Models	**Hazard Ratio on Treatment**	
	**HR [95% CI]**	**P value**
**Subgroup**		
Glucose in the bottom quartile	1.11 [0.67-1.82]	0.698
Urine albumin/creatinine ratio in bottom half	0.54 [0.35-0.83]	0.005
Glucose in the bottom quartile & Urine albumin/creatinine ratio in bottom half	3.17 [0.96-10.42]	0.058

Panel B. Results of Interaction Models	**Hazard Ratio on Interaction Term**	
	**HR [95% CI]**	**P value**
**2-Way Interaction Model: Glucose**		
Glucose in the bottom quartile × Treatment	1.77 [1.02-3.06]	0.043
**2-Way Interaction Model: Urine albumin/creatinine ratio**		
Urine albumin/creatinine ratio in bottom half × Treatment	0.61 [0.36-1.01]	0.054
**3-Way Interaction Model**		
Glucose in the bottom quartile × Treatment	1.05 [0.54-2.03]	0.884
Urine albumin/creatinine ratio in bottom half × Treatment	0.42 [0.23-0.77]	0.005
Glucose in the bottom quartile × Urine albumin/creatinine ratio in bottom half × Treatment	5.66 [1.58-20.22]	0.008

© 2019 Scarpa J et al. *JAMA Network Open*.

eFigure 1. Blood Pressure Changes Across Treatment Group for Hypertensive Smokers vs. Remaining Participations

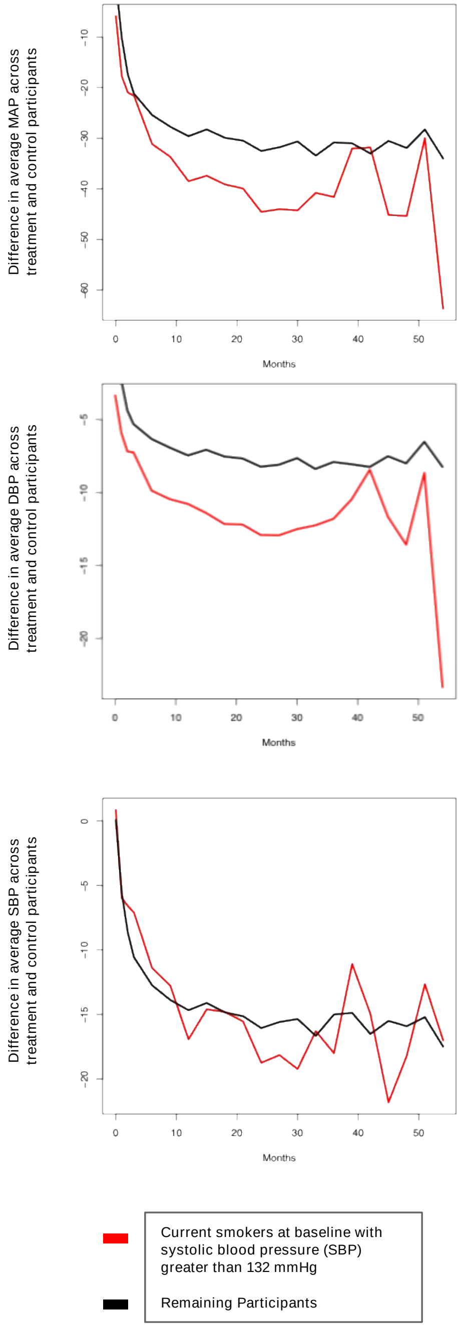

These plots show the difference across treatment and control participants in average blood pressure measurements Mean Arterial Pressure (MAP), Diastolic Blood Pressure (DBP), and

© 2019 Scarpa J et al. *JAMA Network Open*.

Systolic Blood Pressure (SBP)), respectively, over time for the baseline hypertensive smoker subgroup vs. remaining participants**.**

© 2019 Scarpa J et al. *JAMA Network Open*.

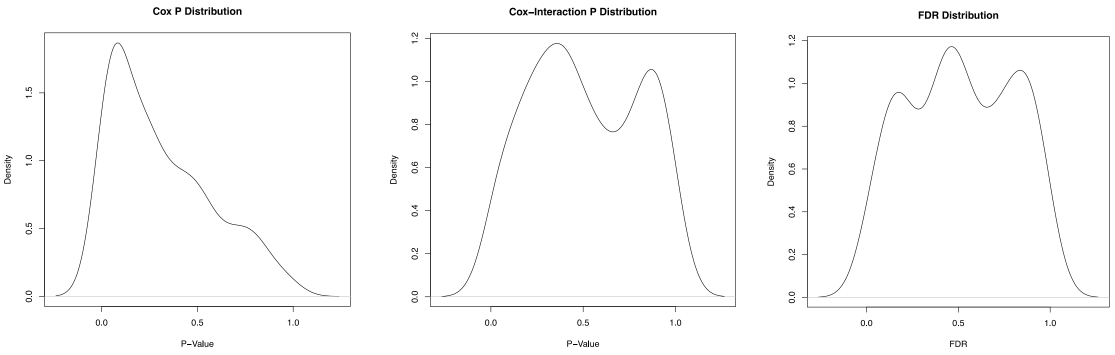

eFigure 2. Distributions of P-values and False Discovery Rates Across Subgroups Identified by the Causal Forest

Left: Distribution of treatment term p-values across subgroups identified by the forest. *Middle:* Distribution of interaction term p-values across subgroups identified by the forest. *Right:* Distribution of FDRs across subgroups identified by the forest.

© 2019 Scarpa J et al. *JAMA Network Open*.

eReferences

1. Group SR. A randomized trial of intensive versus standard blood-pressure control. N Engl J Med. 2015;2015(373):2103-16.

2. Willke RJ, Zheng Z, Subedi P, Althin R, Mullins CD. From concepts, theory, and evidence of heterogeneity of treatment effects to methodological approaches: a primer. BMC Med Res Methodol. 2012;12:185.

3. Athey S, Imbens G. Recursive partitioning for heterogeneous causal effects. Proc Natl Acad Sci U S A. 2016;113(27):7353-60.

4. Breiman L, Friedman J, Stone CJ, RA O. Classification and regression trees. Boca Raton, FL: CRC press; 1984.

5. Breiman L. Random forests. Machine Learning. 2001;45(1):5-32.

© 2019 Scarpa J et al. *JAMA Network Open*.
